# Hyperthermia-Triggered Gemcitabine Release from Polymer-Coated Magnetite Nanoparticles

**DOI:** 10.3390/polym10030269

**Published:** 2018-03-06

**Authors:** G. R. Iglesias, Felisa Reyes-Ortega, B. L. Checa Fernandez, Ángel V. Delgado

**Affiliations:** Department of Applied Physics, Facultad de Ciencias, Campus Fuentenueva, School of Sciences, and MNAT Unit of Excellence, University of Granada, 18071 Granada, Spain; felisareyes@ugr.es (F.R.-O.); lunachecaf@gmail.com (B.L.C.F.); adelgado@ugr.es (A.V.D.)

**Keywords:** biocompatible polymer, drug delivery, gemcitabine, magnetic hyperthermia, magnetic nanoparticles

## Abstract

In this work a combined, multifunctional platform, which was devised for the simultaneous application of magnetic hyperthermia and the delivery of the antitumor drug gemcitabine, is described and tested in vitro. The system consists of magnetite particles embedded in a polymer envelope, designed to make them biocompatible, thanks to the presence of poly (ethylene glycol) in the polymer shell. The commercial particles, after thorough cleaning, are provided with carboxyl terminal groups, so that at physiological pH they present negative surface charge. This was proved by electrophoresis, and makes it possible to electrostatically adsorb gemcitabine hydrochloride, which is the active drug of the resulting nanostructure. Both electrophoresis and infrared spectroscopy are used to confirm the adsorption of the drug. The gemcitabine-loaded particles are tested regarding their ability to release it while heating the surroundings by magnetic hyperthermia, in principle their chances as antitumor agents. The release, with first-order kinetics, is found to be faster when carried out in a thermostated bath at 43 °C than at 37 °C, as expected. But, the main result of this investigation is that while the particles retain their hyperthermia response, with reasonably high heating power, they release the drug faster and with zeroth-order kinetics when they are maintained at 43 °C under the action of the alternating magnetic field used for hyperthermia.

## 1. Introduction

The interest of nanoparticle (NP) science and technology spreads over a large number of fields, biomedicine being one where applications are becoming closest to everyday life [1,2,3,4,5,6]. The wide range of nanostructures built with that purpose, the practically unlimited functionalization routes, and, of course, the correct size scale in comparison with cells and their membranes may partially account for an explanation to these facts. Among the many nanoparticles designs, either organic, inorganic, or mixed, those that are based on magnetic iron oxides (mostly magnetite and maghemite), hence being called magnetic nanoparticles (MNPs), have seen their possibilities multiplied because of their magnetic response. It is specifically desired to have a paramagnetic-like behavior, so that the particles are only magnetized in the presence of the field, with no remanence or coercivity. They are then denominated SPIONs (superparamagnetic iron oxide nanoparticles), and they have been proposed to be part of disease diagnose and treatment (or theranostics), very particularly in the case of solid tumors [7,8,9,10,11,12,13,14,15,16,17]. It is precisely their response to either dc or ac magnetic fields that determines the fields of biomedical applications of MNPs. One of them is drug delivery: the particles would be part of so-called magnetic vectors, which are loaded with a chemotherapeutic drug and functionalized in such a way that they can mostly escape from the cells of the immune system, in addition to being able to specifically interact with the target tumor cells. A non-homogeneous, externally applied magnetic field can be used to drive the particles to their site of action, or, at least, keep them there after being transported by the blood stream [11,18,19,20,21,22]. Many in vitro and in vivo tests have demonstrated the feasibility of this approach, but no formulation based on MNPs for drug delivery has been approved to date. In contrast, Drug Agencies have approved formulations based on MNPs, to be used as contrast agents in magnetic resonance imaging [23,24,25,26].

A very promising application field is that of magnetic hyperthermia. When a suspension of magnetic particles is subjected to an alternating magnetic field of suitable frequency and strength, heat is released which will warm up the suspension and its surroundings. If applied to particles that are embedded in a tumor, and if the temperature is raised to 42–43 °C for at least half an hour, the heating will produce the death of the tumor cells (healthy ones can withstand this temperature safely), and eventually provoke the disappearance of the tumor or at least help other therapeutic approaches in that task [27,28,29,30,31,32,33,34,35,36,37]. The mechanism by which the heating occurs depends on the particle size: in the case of SPIONs, both eddy currents and hysteresis losses can be neglected, and only Néel and/or Brownian (or viscous) relaxations are possible [28,36,38,39]: the former refer to the oscillations of the magnetic moment of the NP back and forth between two easy directions of magnetization, in an otherwise immobile particle. The second mechanism involves the rotational motion of the particle itself in the suspending fluid. Both of the processes can be present at a time, depending on the frequency-size combination [36,40], and in the two of them there is a lag between magnetization and field (that is, an imaginary component of the susceptibility), ultimately being responsible for the heat loss.

There is indeed the possibility of designing MNPs aimed at being used in more than one of the cited applications. For instance, contrast in MRI can help in locating the distribution of magnetic particles while applying hyperthermia [41,42,43,44]. Closer to the objectives of the present work is the use of the magnetic particles in nanostructures that are aimed at the simultaneous application of hyperthermia and drug delivery in-situ, an application denominated thermotherapy by some authors [45]. The idea is double: the vehicles can deliver the drug while producing magnetic hyperthermia, and, furthermore, the latter can trigger or speed up the drug release. It has been shown to work as expected in a number of combinations, for instance doxorubicin on different magnetic substrates, including magnetite/thermoresponsive polymer [46,47,48], magnetite/phospholipid/poly (etilenglicol) [49], or magnetite/poly(ethylene imine)/poly(styrene sulfonate)/PEG [50], to mention a few.

In this paper, the commercial magnetic particles used consist of a magnetite core and a polymeric envelope, a co-polymer of methyl methacrylate/ethylene glycol dimethacrylate/hydroxyl ethyl methacrylate. We describe the procedure that is used for transforming them into suitable substrates for the electrostatic adsorption of gemcitabine (GEM). This is an antitumor agent that is employed extensively against several human cancers [51,52,53,54,55,56], including ovarian, lung, pancreatic, bladder, urothelial, and breast cancer. Furthermore, this drug has been approved by FDA [57] and it is the most prescribed anticancer drug worldwide, alone or in combination with other chemotherapeutic agents, namely doxorubicin [58,59]. While the latter has been widely studied as a single antitumor agent, more scarce investigations have been carried out with gemcitabine. For this reason, this work has focused on this drug, in an attempt to broaden our understanding of the delivery mechanism of this drug. The biocompatible polymer-coated MNPs were loaded with GEM and their release rate investigated in three cases: suspensions kept in a thermostated bath at 37 °C or 43 °C, or were subjected to magnetic hyperthermia at 43 °C for specified time intervals. It appears that the latter brings about a substantial improvement in the drug release rate, and this constitutes a promising result regarding the design of multi-functional platforms for cancer therapy.

## 2. Materials and Methods

### 2.1. Materials

**Scheme 1:**
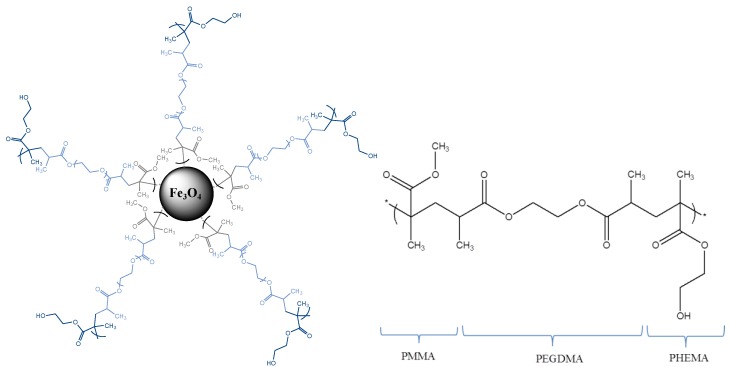
Chemical structure of the MagP^®^–OH magnetic nanoparticles.

MagP^®^–OH particles were purchased from NanoMyP (Granada, Spain). According to the manufacturer, these composite particles contain magnetite NPs that are coated with a polymer shell (Scheme 1) composed of 54 wt % methyl methacrylate, 31 wt % ethylene glycol dimethacrylate, and 15 wt %, hydroxyl ethyl methacrylate, or poly(MMA-*co*-EGDMA-*co*-HEMA). The terminal monomers are MMA (bound to the particles by emulsion polymerization), and HEMA (providing surface OH groups to the composites). The EGDMA polymer acts as crosslinker and determines the thickness of the layer and adds biocompatibility to the particles, as PEG is recognized as one of the best options for avoiding opsonization and subsequent attack to the particles by the cells of the mononuclear phagocyte system [49,60,61]. The original particles are coated with a surfactant (SDS) in order to facilitate their stability. This layer was eliminated to the maximum possible extent by repeated centrifugation at 14,000× rpm and redispersion in water.

*N*-hydroxysuccinimide (NHS) (98%), 4-(dimethylamino) pyridine (DMAP) (99%), dichloromethane anhydrous (DCM), and phosphate buffered saline (PBS) tablets were purchased from Sigma-Aldrich (St. Lois, MO, USA). Gemcitabine hydrochloride was acquired from Adooq Bioscience LLC (Irvine, CA, USA). Water that was used in the preparation of the solutions was deionized in a Milli-Q Academic (Madrid, Spain) device, with conductivity of 0.054 μS/cm at 25 °C.

### 2.2. Methods

#### 2.2.1. Functionalization with –COOH Terminal Groups

In order to increase the surface charge of the MagP^®^–OH at physiological pH, thus being likely improving the ionic attachment, 2 equivalents of NHS (5.3 mg), 1 equivalent of MagP^®^–OH (100 mg) and 1 equivalent of DMAP (2.8 mg) were added to a flask that was previously purged with nitrogen. Then, 20 mL of dry DCM was added under the nitrogen atmosphere, and the mixture was mechanically stirred for 24 h at room temperature. Afterwards, the particles were magnetically decanted and washed several times with a mixture of ethanol and distilled water (1:1), and finally dried at 60 °C in an oven. The resulting particles will have terminal carboxylic groups (and hence will be denominated MagP–COOH hereafter) and hence higher charge surface than the particles with terminal hydroxyl groups, allowing for higher ionic attachment efficiency with positive charged drugs.

#### 2.2.2. Adsorption of Gemcitabine to MagP–COOH

GEM was incorporated to the MagP–COOH particles by dispersing them in an aqueous solution of the drug. Concisely, different amounts of MagP–COOH were dispersed into 1 mL of an aqueous 1 mM GEM solution, under mechanical stirring at room temperature, for 18 h. Then, the particles with the adsorbed GEM were centrifuged at 14,000× rpm for 30 min to remove the supernatant. The same procedure was used for the repeated washing of the particles with Milli-Q water. The final step consisted of freeze-drying the GEM-coated particles in order to obtain the loaded magnetic vectors.

#### 2.2.3. Morphology and Size Distribution

The morphology of the nanoparticles was observed by transmission electron microscopy (TEM) using a LIBRA 120 Plus Carl Zeiss microscope (Oberkochen, Germany). TEM images were analyzed with J-Image software in order to calculate the particle size distribution of the dried NPs. These results were compared with data obtained by DLS, using a Nano-ZS apparatus (Malvern Instruments, Worcestershire, UK). All of the measurements were performed at 1 mg/L in Milli-Q water at 25 °C.

#### 2.2.4. Electrophoretic Mobility and Zeta Potential

Electrophoretic mobility measurements were carried out in the Nano-ZS at 25 °C, in suspensions with about 0.01% *w*/*v* solids concentration and a constant ionic strength of 5 mM KNO_3_. The pH value of the suspensions was then adjusted by adding a suitable amount of KOH (0.01 or 0.1 M) or HNO_3_ (0.01 or 0.1 M). For each suspension, five measurement runs were taken, with 11 cycles in each run. The zeta potential, *ξ*, was obtained from the O’Brien and White electrophoresis theory [60].

#### 2.2.5. ATR-FTIR Characterization

FTIR spectra were obtained in the Attenuated Total Reflection mode (ATR-FTIR) in a JASCO 6200 FT-IR (Tokyo, Japan) spectrometer with SPECTRA MANAGER V2 software. Samples were analyzed without further treatment at room temperature with 50 scans and a resolution of 4 cm^−1^.

#### 2.2.6. Thermogravimetric Analysis

Thermogravimetric analysis (TGA) was performed on a TGA-50H SHIMADZU (Kyoto, Japan) device with vertical oven and a maximum precision of 0.001 mg. Samples were analyzed under 50 mL/min nitrogen flow, and at a heating rate of 10 °C/min, from 30 to 900 °C.

#### 2.2.7. Magnetic Properties

Magnetization cycles were obtained at room temperature in an MPMS-XL SQUID magnetometer (Quantum Design, San Diego, CA, USA).

#### 2.2.8. Magnetic Hyperthermia and Specific Absorption Rate Determination

Magnetic hyperthermia was implemented using an AC current generator built with a Royer-type oscillator. An eight turn coil (20 mm in diameter and 45 mm in length) that was made of 6 mm water cooled copper tube was connected to the oscillator in parallel with different combinations of capacitors, so that the field frequencies were changed, with fixed values of 185, 206, 236, and 285 kHz. The current through the coil (typically 7 A) was selected to reach a magnetic field amplitude of 16.2 kA/m (20 mT magnetic induction in air), measured at the center of the coil with a NanoScience Laboratories Ltd., Probe (Newcastle, UK), with 10 μT resolution.

The hyperthermia efficiency of the particles was quantified by measuring the rate of temperature increase registered with an optical fiber thermometer (Optocon AG, Dresden, Germany). The fiber probe was placed into a 5 mL Eppendorf tube containing 0.5 mL of a 10 mg/mL suspension of the magnetic nanoparticles, previously pre-thermostated at 37 °C; this was also the temperature that was chosen for the water circulating inside the copper coil. The tube was in turn inserted in the coil, and was thermally insulated from it by using a Styrofoam chamber. From the initial slope of the temperature vs. time dependence (typically, the first 30 s after switching the field on), *dT*/*dt*, the specific absorption rate, *SAR*, was obtained, as follows [28,62]:

(1) $$SAR=\frac{CV_{s}}{m}\frac{dT}{dt}$$

where *C* is the volume specific heat capacity of the sample ($C_{H_{2}O}=$ 4185 J/LK), *V_s_* is the sample volume (0.5 mL in the reported experiments), and *m* is the mass of solids in the sample (5 mg). If a linear relationship between the sample magnetization and the field is assumed (a reasonable hypothesis for the small field amplitude used), the power density (per unit volume of particles) of heating can be calculated as:

(2) $$\rho_{W}=\mu_{0}\pi f\chi^{\prime \prime }H_{0}^{2}$$

in terms of the imaginary component of the magnetic susceptibility, $\chi^{\prime \prime }$, of the particles [27,28,36,63,64]. Hence, the *SAR* is found to depend on the square of the magnetic field and its frequency. For this reason, some authors prefer to describe the phenomenon for a given sample in terms of the so-called intrinsic loss power or *ILP* [29], given by:

(3) $$ILP=\frac{SAR}{fH_{0}^{2}}$$

Typically, *ILP* is given in units of nHm^2^/kg (*H*_0_ in kA/m, *f* in kHz, and *SAR* in W/kg).

#### 2.2.9. Drug Loading and Release

The drug adsorption on the particles was evaluated by means of optical absorbance determinations at 268 nm wavelength in a 6705 UV/Vis JENWAY spectrophotometer (Staffordshire, UK). The amount of non-adsorbed drug recovered in the external aqueous phase after centrifugation was quantified using a calibration line that was obtained from absorbance measurements in solutions of different concentrations of GEM in PBS. The drug release from loaded particles was also determined spectrophotometrically, as follows: 20 mg of particles was dispersed in 5 mL of PBS (pH 7.4); at specified time intervals, particles were magnetically decanted, then 0.5 mL of the supernatant was removed and replaced by the same volume of pure PBS. The removed aliquot was then centrifuged at 14,000× rpm for 30 min at room temperature, the absorbance of the supernatant was measured, and the amount of GEM was calculated from the calibration line. This allowed for calculating the amount of GEM released. Experiments were first run in the absence of any applied magnetic field, at two temperatures: 37 °C (physiological) and 43 °C (end temperature set in the hyperthermia experiments). The release study with the AC magnetic field applied was similarly carried out, but controlling the magnetic field strength to ensure a constant temperature of 43.0 ± 0.5 °C.

## 3. Results and Discussion

### 3.1. Morphology and Particle Size Distribution

**Figure 1:**
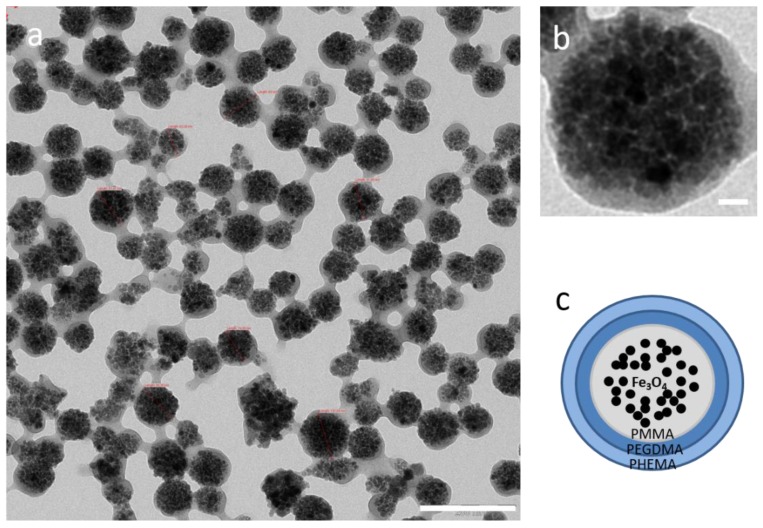
(**a**) TEM image of MagP^®^–OH particles. Scale bar: 200 nm. (**b**) Detail of a single particle. Scale bar is 20 nm. (**c**) MagP^®^–OH particle scheme.

MagP^®^–OH particles were observed in the TEM after surfactant removal. As shown in Figure 1, they can be described as a polymer envelope encapsulating the magnetite cores. 

**Figure 2:**
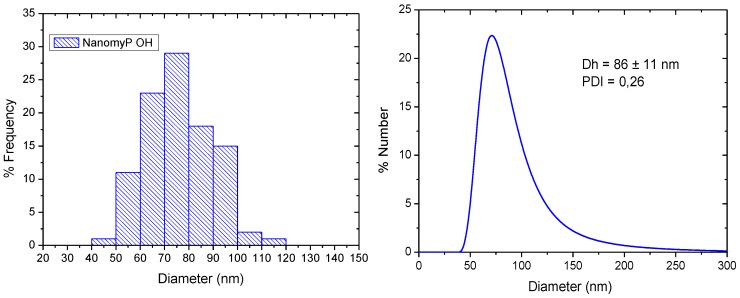
Left: Diameter histogram of the MagP^®^–OH particles calculated from TEM images. Right: size distribution by number, calculated from DLS data.

The histogram of the particle size distribution deduced from the TEM images (Figure 2a), reveals a mean (±SD) particle diameter of 76 ± 13 nm. The hydrodynamic diameter *D_h_* of these particles when they were dispersed in water was measured by DLS and the resulting size distribution by number is shown in Figure 2b; from this, the mean diameter results *D_h_* = 86 ± 11 nm with a polydispersity index (PDI) of 0.26. As it happens usually, the hydrodynamic diameter is somewhat higher than that obtained by TEM due to the hydration of the hydrophilic outmost polymer layer, and to the possibility of some extent of particle aggregation in the aqueous medium.

### 3.2. Zeta Potential

**Figure 3:**
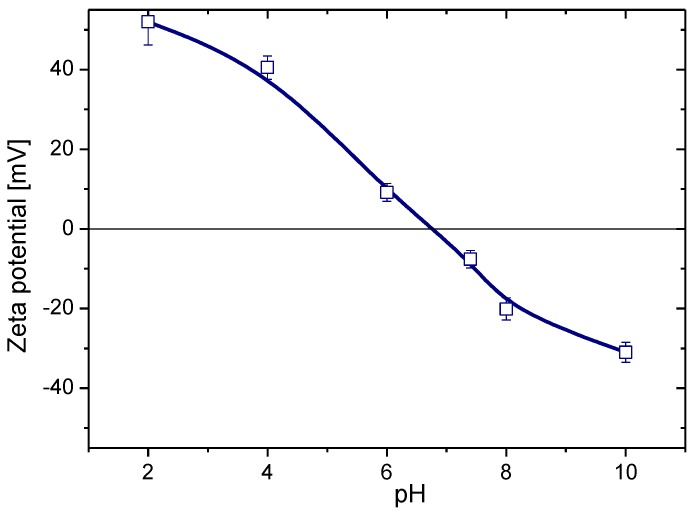
Zeta potential of MagP^®^–OH versus pH in 5 mM KNO_3_.

The commercial MagP^®^–OH NPs showed a negative zeta potential of −27.7 ± 0.5 mV, before being washed to remove the SDS surfactant. After such removal, the zeta potential was measured at different pHs to determine the surface charge of the particles without the surfactant influence. As it is shown in Figure 3, the isoelectric point of MagP^®^–OH was obtained at pH 6.9. This characterization allows us to select the optimal pH value to carry out the electrostatic attachment of the drug, since the form used, GEM hydrochloride is positively charged and stable at any pH between that corresponding to the $pK_{a}(=3.6)$ of GEM, and pH 10, above which the drug undergoes degradation [65,66]. Therefore, further drug loading and release experiments were carried out at physiological pH, whereby magnetite is negatively charged and the drug is positive and quite stable.

**Figure 4:**
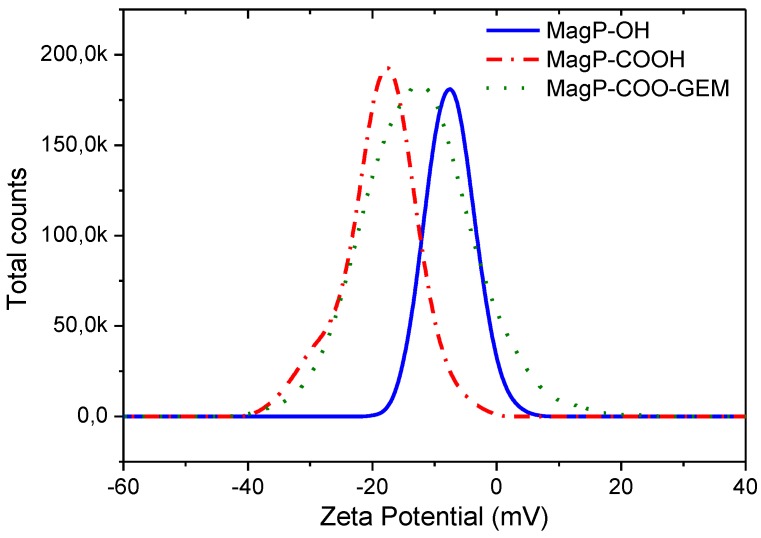
Zeta potential distribution of MagP–OH, MagP–COOH and Gemcitabine loaded MagP–COOH systems.

Figure 4 shows the zeta potential distribution of the commercial NPs (MagP^®^–OH) before and after the carboxylation of the particles (MagP^®^–COOH). It is observed that the zeta potential average at physiological pH becomes more negative (from −7.6 ± 0.2 mV to −17.5 ± 0.3 mV) after carboxylation, and this will promote a higher ionic attachment of the drug. This sort of loading is confirmed by the zeta potential becoming less negative (−12.4 ± 0.3) mV after contact of the MagP^®^–COOH particles (23 mg) with 1 mL of GEM 1 mM aqueous solution, under mechanical stirring for 18 h.

### 3.3. Drug Loading

As mentioned, from the absorbance of the supernatants of the GEM solutions containing suitable amounts of particles, the amount of drug loaded on the particles could be evaluated. Different GEM/MNPs ratios were tested and the results in Table 1 show that the amount of adsorbed GEM when the ratio is 33.3 mg/g was 13.9%, which means 14.3 µM drug/g MNPs. This loading is similar or even higher than those that were reported in the literature regarding incorporation of this drug onto different polymeric NPs [67,68,69]. It is worth mentioning that increasing the initial drug/MNPs ration does not improve the amount of GEM adsorbed (Table 1), which indicates that some sort of electrostatic saturation binding was reached for the concentrations studied. Therefore, the optimal ratio of 33.3 mg GEM/g MNPs was used for further characterization and release studies.

### 3.4. ATR-FTIR Characterization

**Figure 5:**
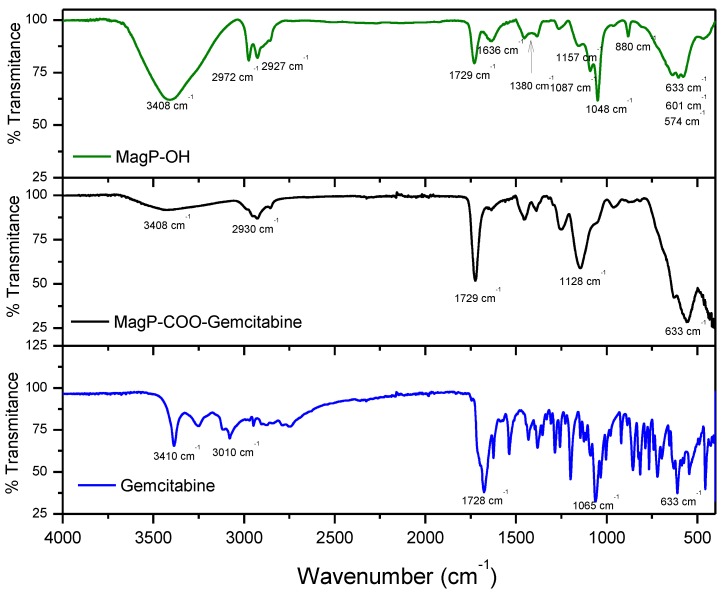
ATR-FTIR spectra of MagP–OH nanoparticles, MagP–COO–GEM nanoparticles, and GEM drug.

Figure 5 shows the infrared spectra of the three kinds of particles that are used in this work (original MagP^®^–OH, GEM and drug-loaded MagP–COO–GEM). Note the presence in the first ones of both magnetite (Fe_3_O_4_) and the methacrylate polymers (PMMA, PEGDMA, PHEMA). The characteristic bands in the range 3200–3600 cm^−1^ are attributed to the stretching of the hydroxyl (OH) groups of the PHEMA component. The bands at 2972 and 2927 cm^−1^ correspond to the C–H stretching vibration belongs to the methyl and methylene groups of the polymers, the bands at 1729 and 1636 cm^−1^ are related to the stretch of the ester carboxyl groups (C=O) of the PMMA, PEGDMA, and PHEMA respectively, the bands to 1452 and 1380 cm^−1^ are attributed to the bending of C–H, the bands at 1264, 1157, and 1087 cm^−1^ belongs to the stretching vibration of the ether groups (C–O) of the methacrylate polymers, band at 1048 cm^−1^ is attributed to the stretching vibration of ether bonds of the polymeric chain belongs to PEGDMA, and the band at 880 cm^−1^ correspond to the single bond deformation vibration of ether groups (C–O). The presence of magnetite is evident due to bands in the range of 570–630 and at 460 cm^−1^, that are due to the stretching vibration bands associated to the iron-oxygen bonds (Fe–O) in the octahedral and tetrahedral sites of the oxide structure.

The ATR-FTIR spectrum of the MagP–COO–GEM shows a noticeable decrease in the –OH band that is situated at 3200–3600 cm^−1^, demonstrating the efficacy of the previous carboxylation of these groups. Besides, the ionic reaction between MagP–COO^−^ and GEM was verified by the increase in the C=O band at 1729 cm^−1^ and the bands modification in the range 500–1500 cm^−1^, where pure GEM shows its characteristic bands.

### 3.5. Thermogravimetric Analysis

**Figure 6:**
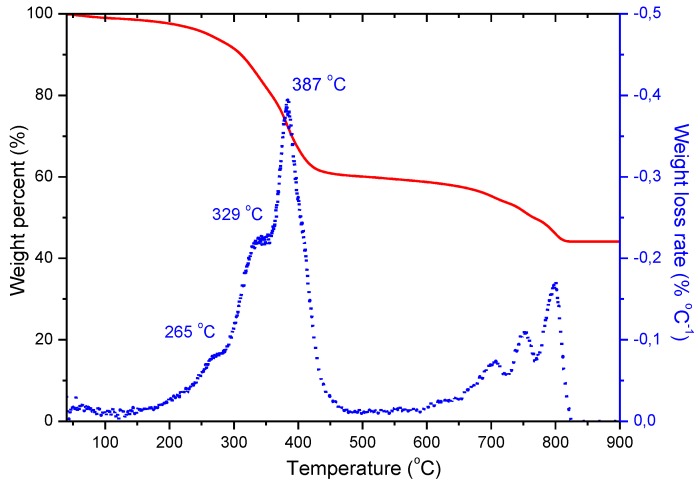
Thermogram of the magnetic nanoparticles.

A thermogravimetric analysis was used to evaluate the thermal stability of the magnetic particles, as well as to check the polymer/magnetite weight ratio and the polymer components. The thermogram of the carboxylated nanoparticles (MagP–COOH) is shown in Figure 6. The weight loss curve shows a total degradation of 41.6 wt % up to 600 °C, which corresponds to the polymeric component of the nanoparticles. The inorganic residue of 58.4% left, according to the TGA analysis corresponds to the magnetite component. Between 600 and 800 °C, the observed weight loss has been associated to magnetite reduction to α-Fe and FeO [70]. The dotted curve shows the weight percentage loss rate versus temperature (first derivative of the weight loss curve), showing three maximum degradation temperatures for the polymeric coating at 265 °C, 329 °C and 387 °C, which correspond to PHEMA, PEGMA, and PMMA respectively [71,72,73]. These degradation steps allow for the quantification of the weight percentage of each polymer in the nanoparticle system, being 11 wt % of PHEMA, 30 wt % of PEGMA, and 59 wt % of PMMA.

### 3.6. Magnetization and Hyperthermia

**Figure 7:**
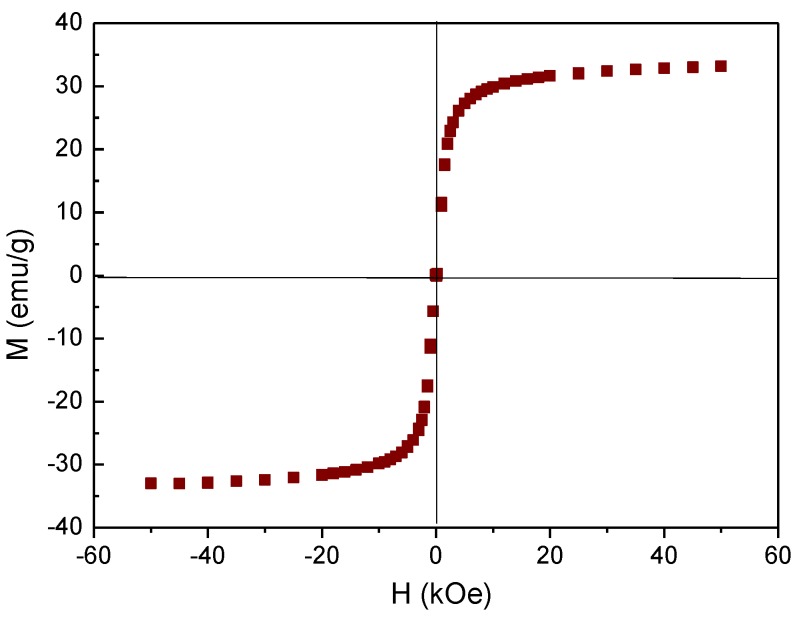
Magnetization curve of MagP^®^–OH magnetic nanoparticles.

The magnetization of MagP^®^–OH NPs as a function of the magnetic field is represented in Figure 7. The superparamagnetic behavior of the core magnetite NPs manifests in the composite nanostructures, although the presence of the non-magnetic polymeric envelope reduces the saturation magnetization from that of bulk magnetite (90 emu/g) to a value close to 35 emu/g. Such kind of a reduction has been previously reported in literature [74,75] and justified by the contribution of the polymer layers and the limited magnetic order at the magnetic NP/polymer interfaces, very abundant in our particles (Figure 1).

**Figure 8:**
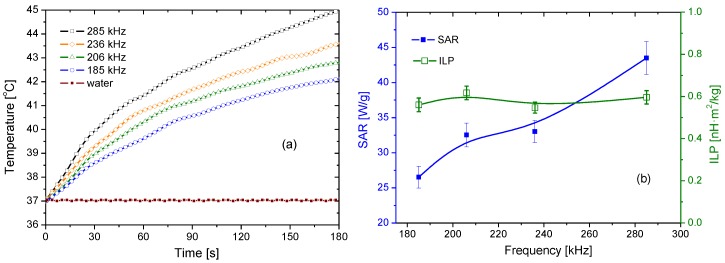
(**a**) Time evolution of the temperature for different frequencies in hyperthermia experiments performed with suspensions containing 10 mg/mL of MagP^®^–OH. (**b**) *SAR* and *ILP* evaluation. The field amplitude was *H*_0_ = 16.2 kA/m in all the cases.

Figure 8a shows the time evolution of the temperature of a suspension of MNPs before loading the drug, for different frequencies of the field (285, 236, 206, and 185 kHz). It is possible to observe that the time needed for reaching 41 °C decreases with frequency. Thus, for the highest frequency, it is found a rapid increase of temperature from 37 to 41 °C in less than a minute, which is ideally desired for cancer treatment application. Pure water baseline is added as reference, showing the negligible effect of Joule heating of the copper coil in the data. The *SAR* and *ILP* values are represented in Figure 8b. Note that the former reaches values that are in the high range of those reported in the literature, and that they increase with the field frequency, as mentioned. On the contrary, the intrinsic loss power is frequency independent, in agreement with the predictions of Equation (3). The analysis of these results suggests that the particles will be a useful tool to produce local heating and generate enough heat as to locally rise the temperature of the tumor tissue for the effective hyperthermia treatment.

### 3.7. Drug Release

**Figure 9:**
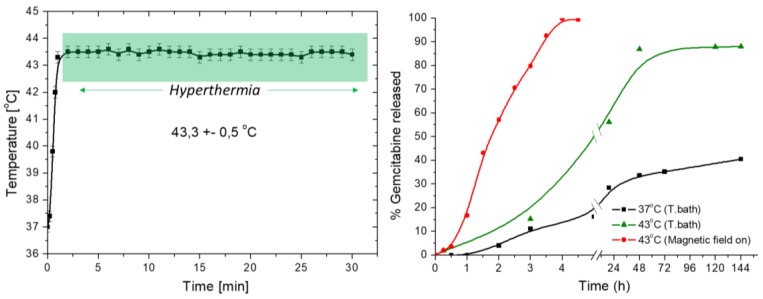
(**a**) Typical hyperthermia and drug release experiment with manually stabilized magnetic field strength; (**b**) GEM release profile for thermostatic bath at 37 °C, 43 °C, and magnetic field hyperthermia set at 43 °C.

GEM release studies were performed in PBS buffer at pH 7.4, which mimics the physiological conditions. Drug release was tested at controlled temperatures of 37 ± 1 °C and 43 ± 1 °C. A third release test was carried out using the AC magnetic field that is applied in hyperthermia, so as to evaluate the potential triggering effect of the field on the release profile of the drug. A typical hyperthermia and drug release experiment is shown in Figure 9. The nanoparticles that are loaded with the drug were exposed to the magnetic field for 30 min [76,77]. After a rapid increase in temperature, the system was stabilized between 43 °C and 44 °C by manually controlling the magnetic field strength. Drug release results are plotted in Figure 9b. As observed, the fastest release profile of GEM was obtained when the magnetic field was applied, allowing for the complete release of the drug in less than 4 h. The kinetic release in this case follows a quasi-zeroth order profile [78], with an initial delay in the drug release, probably due to the time needed to reach the optimal triggering temperature. The released amount *R*(*t*) can be fitted to the Equation (4):

(4) $$R(t)=R(0)+k_{0}t$$

where *R*(0) is the initial amount of drug in the solution and *k*_0_ is the zeroth-order release constant expressed in units of concentration/time, which was found to be 27 µg/mL·h. This kinetic constant is much higher than values that are reported in literature for GEM thermal release [79], demonstrating the improvement of the GEM release with the application of the AC field.

In fact, the release of GEM at 43 °C (temperature controlled without field applied) showed a faster release profile than at 37 °C, reaching a plateau of 90% of the drug after 48 h, when compared to the 35% released at 37 °C after the same time. Only 40% of the total drug was released at 37 °C after six days. Longer time is needed to release the complete amount of drug at physiological temperature, resulting in a negligible release rate. The release dependences found at both 37 °C and 43 °C are well described by first order kinetics, according to Equation (5):

(5) $$R(0)-R(t)=R(0)(1-e^{-k_{1}t})$$

where *k*_1_ is the first order rate constant. Its best fit values are 0.092 h^−1^ and 0.056 h^−1^ for 37 and 43 °C, respectively. Note that in a first order release profile the process is directly proportional to the drug concentration that is involved in the process, whereas in a zeroth-order release the drug is released at a constant rate, leading to the best control of plasma concentration and offering several advantages, including improved patient compliance and reduction in the frequency of drug administration [80,81]. This feature is also a positive result of using hyperthermia in combination with drug release.

## 4. Conclusions

The combination of drug release and magnetic hyperthermia using magnetic nanoparticles consisting of a magnetite core and a polymer envelope is investigated for modified commercial particles in which the –OH terminated co-polymer is modified to make it –COOH terminated. The particles are found to be quite monodisperse in size and spherical in shape, with negative zeta potential in physiological pH conditions. They showed a very significant hyperthermia response, with *SAR* values of up to 45 W/g. Gemcitabine hydrochloride was absorbed by contact in solution between the particles and the drug. The loading was optimal at a rate of 33.3 mg GEM/g MNPs, and is demonstrated by IR spectrometry and electrophoresis. The drug release in PBS at pH 7.4 was investigated in three experimental conditions: temperature that was maintained at 37 °C or 43 °C in a temperature controlled bath, and hyperthermia-triggered release at 43 °C. It is demonstrated that drug delivery in the latter case proceeds at zeroth order kinetics and the magnetic field exposure triggers and improves the release significantly, reaching 100% delivery in less than 4 h.

## Figures and Tables

**Table 1:** GEM adsorbed on MagP–COOH NPs for different initial drug/MNPs ratios.

Initial Drug/MNPs Ratio (mg/g)	Adsorbed GEM (mg/g MNPs)	Fraction of Adsorbed Drug (%)
33.3	4.3	13.9
66.7	4.0	6.0
100	3.0	3.0
